# Study of the Failure Mechanism of a High-Density Polyethylene Liner in a Type IV High-Pressure Storage Tank

**DOI:** 10.3390/polym16060779

**Published:** 2024-03-12

**Authors:** Alfredo Rondinella, Giovanni Capurso, Matteo Zanocco, Federico Basso, Chiara Calligaro, Davide Menotti, Alberto Agnoletti, Lorenzo Fedrizzi

**Affiliations:** 1 Polytechnic Department of Engineering and Architecture, University of Udine, Via del Cotonificio 108, 33100 Udine, Italy; matteo.zanocco@uniud.it (M.Z.); lorenzo.fedrizzi@uniud.it (L.F.); 2 Department of Agricultural, Food, Environmental, and Animal Sciences, University of Udine, Via Sondrio 2/A, 33100 Udine, Italy; federico.basso@uniud.it; 3 Faber Industrie SpA, Via dell’Industria 64, 33043 Cividale del Friuli, Italy

**Keywords:** HDPE, failure analysis, polymer degradation, high-pressure storage tanks

## Abstract

The use of Type IV cylinders for gas storage is becoming more widespread in various sectors, especially in transportation, owing to the lightweight nature of this type of cylinder, which is composed of a polymeric liner that exerts a barrier effect and an outer composite material shell that primarily imparts mechanical strength. In this work, the failure analysis of an HDPE liner in a Type IV cylinder for high-pressure storage was carried out. The breakdown occurred during a cyclic pressure test at room temperature and manifested in the hemispherical head area, as cracks perpendicular to the liner pinch-off line. The failed sample was thoroughly investigated and its characteristics were compared with those of other liners at different stages of production of a Type IV cylinder (blow molding, curing of the composite material). An examination of the liner showed that no significant chemical and morphological changes occurred during the production cycle of a Type IV cylinder that could justify the liner rupture, and that the most likely cause of failure was a design-related fatigue phenomenon.

## 1. Introduction

One of the biggest challenges facing a transition towards a sustainable energy future is filling the gap (both in the time and in space) between renewable production and use; hence, an infrastructure for energy storage should be implemented. To this end, the key factor, which needs to be developed before fully employing hydrogen as an energy carrier, is an optimal way to handle and store H_2_ [1]. Although the conversion of renewable energies into this vector has many advantages (for example the high gravimetric energy density), and it surely represents a promising solution for a transition to a more sustainable scenario, there are few drawbacks, due to the low volumetric energy density of hydrogen. Therefore, in order to increase this value, several physical and chemical hydrogen storage methods were developed, such as pressurized gas, cryogenic liquid, and solid-state systems as chemical or physical combinations with materials [2,3,4].

Pressurized storage is the most common technique and it has already reached a high technology-readiness level, although the capacity to reach high pressures needs to be attained before it can be used effectively [5,6]. Despite the work required to compress the gas and some mild safety concerns, this technology is mature and widespread with several commercial solutions [1]. In order to maximize the efficiency and contain the costs of storing compressed hydrogen in automotive applications, materials should be lightweight while maintaining mechanical performance [7,8]. For this reason, although different types of cylinders are used for the storage of pressurized hydrogen, one of the most popular solutions is the use of Type IV storage tanks. Unlike the previous generations of tanks, that is to say, Type I to Type III, where the first barrier for the gas is always a metallic material, they are composed of a thermoplastic polymer liner [9,10] assembled with a metal boss and wrapped with a thermoset matrix composite material, such as a carbon or glass-fiber-reinforced epoxy resin [11,12,13].

The composite material shell gives the cylinder its mechanical strength, while the thermoplastic liner exerts a barrier effect against the diffusion of gas [14,15]. Even though the liner can be produced with different thermoplastic polymers, such as polyurethane and polyamides [16], polyethylene still remains the most popular material choice to manufacture this component [17,18,19]. These liners are subjected to several filling and emptying cycles, which can induce not only a thermal gradient, generated by the compression and expansion of hydrogen [15], but also a hydrostatic load in the composite-confined liner [15]. During loading and unloading cycles, the liner is subjected to very high pressure variations. In fact, it can go from relatively low pressures, of about 20 bar, to very high values of up to 875 bar [13]. Depending on the operating conditions, these stresses can lead to several degradation phenomena and to some failure cases that are still being studied and modeled today.

Several cases of polymer liner failure are already reported in the scientific literature [20]. Collapse is usually due to a combination of several degradation phenomena that are closely intertwined due to the polymeric nature of the liner. The combined effect of fatigue cycles and temperature changes can in fact amplify the effects of stresses acting on the polymer and cause both a change in gas permeability [2,21] and thermo-mechanical degradation phenomena [22,23,24]. Therefore, temperature and pressure cycling can lead to two significant issues. First, it can lead to unwanted gas permeation that can cause the formation of voids, bubbles, dilation, or blistering [15,25,26,27] and the gas to be out of pressure equilibrium. However, it can also cause a substantial decrease in the mechanical performance of the liner [28]. Alterations in the physical and chemical properties of high-density polyethylene (HDPE), due to rapid hydrogen gas release have been observed, too [29]. In this instance, the overall crystallinity was reduced, and both hydrogenation and oxidation occurred, adding atoms to macromolecular chains and forming carbonyl in ester groups, respectively; increases in methyl group terminations and crosslinking were observed, as well [29].

For these reasons, research is constantly focused on identifying and comprehending the degradation mechanisms of these types of materials and preventing them [15,28,30]. Combining various chemical and morphological analysis techniques, the present research studies a particular plastic deformation that occurs near the parison pinch-off seam of the mold parting line of a blow molded HDPE liner. The research aims to understand the possible causes that led to a phenomenon with a yet undocumented geometry of failure in a liner, during a pressure cycling test.

## 2. Materials and Methods

### 2.1. HDPE Liner

The HDPE liner studied during the failure analysis is a cylindrical container with a capacity of 40.6 L, produced by blow molding. The liner is normally wrapped by the composite material, made of carbon fiber reinforced epoxy matrix, through a filament winding process. The resulting cylinder is then thermally cured in an oven with a specific temperature-time profile.

These types of cylinders are then subjected to a hydrostatic test at a pressure prescribed by the reference standard [31,32] and then fatigue tested for long-term leakage. The test that caused the liner failure under examination is a hydraulic-pressure cycle test performed at room temperature. The pressure is varied from a minimum value of about 20 bar to a maximum value of 525 bar following the requirements of ISO 11119-part 3 [32]. The liner showed failure below the 12,000 cycle-threshold required to pass the test.

Following the failure, the composite material was removed from the cylinder and a visual inspection was conducted to locate the point of failure. Samples acquired from the liner investigated in this failure analysis will be indicated as “Failed”.

In addition to the sample that broke down during testing, several others, taken during different stages of liner production, were analyzed and are briefly described below:

- “Blow molded”—the liner taken just after the molding process;
- “Cured”—the liner after the entire cylinder has been placed in an autoclave to promote composite material curing. This liner was taken from the normal production line just after curing.

### 2.2. Sample Characterization

The liners were cut into smaller samples and characterized by means of scanning electron microscopy (SEM) using a ZEISS EVO 40 microscope (Carl Zeiss Microscopy GmbH, Oberkochen, Germany), equipped with Energy Dispersive X-ray Spectroscopy (EDXS), to investigate the morphology and chemistry of the failed surface and of the cross section. The samples were coated with a thin layer of Au with a Sputter Coater 108 auto (Cressington Scientific Instruments Ltd., Watford, UK) to avoid image distortion due to a charging effect on the non-conductive surface of the observed pieces. In the case of the specimen to be observed in cross-section, the investigated portions were embedded in slow-curing transparent epoxy (Epofix from Streuers ApS, Ballerup, Denmark) to facilitate handling and polishing; this mounting process does not affect the polymer properties, because the reticulation occurs at room temperature. Samples were polished with a Forcipol (Metkon Instruments Inc., Bursa, Turkey), with silicon carbide abrasive paper, grit size 2000.

Samples were also analyzed by mean of ATR-FTIR analysis, to further investigate the surface chemical composition. A Nicolet iS50 Fourier transform infrared (FTIR) spectrometer (Thermo Fisher Scientific, Waltham, MA, USA) was utilized with the Attenuated Total Reflection (ATR) technique. Each IR spectrum was obtained with a 2 cm^−1^ resolution within a spectral range spanning from 450 to 4000 cm^−1^, with 32 scans per spectrum.

In order to perform X-ray diffraction (XRD) measurements on the three different samples considered, small portions with a thickness of about 2 mm were also prepared, to fit on the sample holders and present a surface for the focused beam. X-ray diffraction patterns were recorded using a step size of 0.02° and a counting time of 40 s per step, in the range 10–80° using a Philips X’Pert diffractometer in Bragg–Brentano geometry with an X′Celerator detector (PANalytical B.V., Almelo, The Netherlands); the settings of voltage and current were, respectively, 40 kV and 40 mA, and Ni-filtered Cu-Kα radiation (λ  =  1.5418 Å) was used. A semi-quantitative estimation of the crystallinity of the polymeric samples was determined using the ratio between the area of the crystalline peaks over the total area under the curve.

From the specimens, cubic-shaped portions with an approximate side size of 3 mm were then cut out, so that they could be placed inside an aluminum crucible. Differential scanning calorimetry (DSC) analyses were performed with a ramp from −90 to 200 °C at a rate of 10 °C min^−1^ in an inert atmosphere (20 mL min^−1^ continuous nitrogen flow) in a DSC 3 STARe System (Mettler-Toledo GmbH, Greifensee, Switzerland). The following parameters were calculated from thermograms, using STARe software ver. 16.10 (Mettler-Toledo GmbH, Greifensee, Switzerland):

- melting enthalpy;
- crystallinity;
- onset temperature;
- peak temperature.

## 3. Results

**Figure 1:**
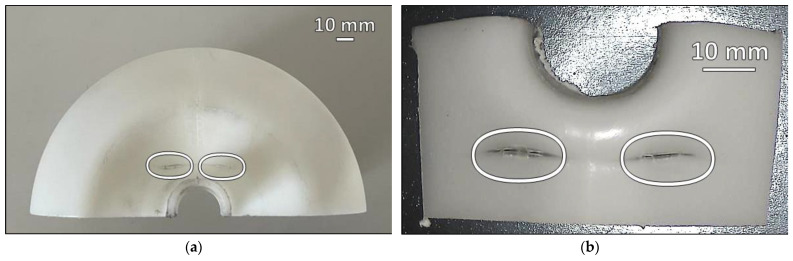
Photographs of the Failed specimen with highlighted failure points: (**a**) half of the hemispherical head area of the failed liner and (**b**) a detail of the cracks as seen from inside the cylinder.

A visual inspection of the Failed specimen revealed cracks perpendicular to the liner mold parting line (pinch-off line) with a length of about 2.5 cm, as visible clearly from above in Figure 1a, where such defects are marked with ovals. In Figure 1b, these cracks are seen from below and highlighted with ovals; their extension, from this perspective, seems to be shorter. The morphology in the two photographs clearly shows plastic deformation and polymeric material pushing outward.

**Figure 2:**
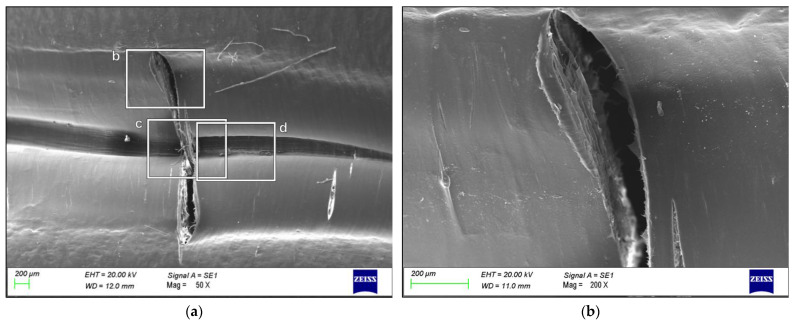

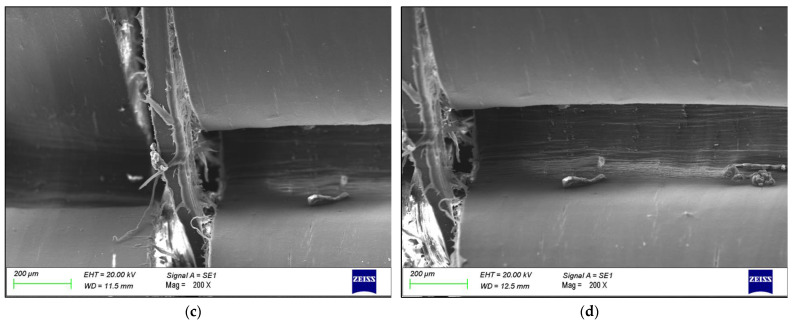
SEM micrographs of (**a**) one of the cracks occurred in the Failed specimen highlighted in the photographs in Figure 1, and details of several areas highlighting: (**b**) the fracture point, (**c**) fiber fraying, and (**d**) a change in surface morphology.

Figure 2 shows more clearly the plastic deformation that occurred during the prescribed cyclic test (already highlighted in Figure 1). Figure 2b, 2c, and 2d underline some of the details of Figure 2a by showing one of the breaking points, polymer fiber fraying and a change in surface morphology, respectively. In particular, looking at the micrographs in Figure 2, it is possible to assume a reorientation of the polymer caused by the phenomenon that led to the rupture.

**Figure 3:**
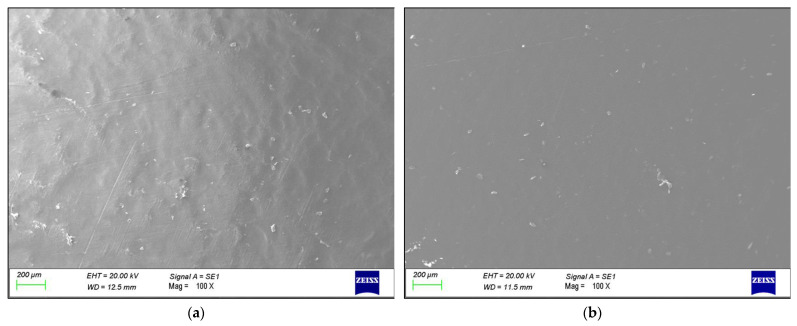
Surface morphology of the polyethylene liner in the (**a**) “Blow Molded” and (**b**) “Cured” samples.

The SEM micrographs in Figure 3 show the surface of the Blow Molded and Cured samples, the reference material before the fatigue tests. Although a slight morphological change of the surface seems to occur from one step to the next, no major imperfections are detectable, suggesting that the manufacturing process does not cause surface changes that would trigger a cracking phenomenon.

A comparison of the results of the EDXS analysis performed on the surface of the specimens throughout the production process (*cf.* Figure 2 and Figure 3) is shown in Table 1 and points to the slight oxidation of the surface following failure. The values shown in Table 1 are averaged from several analyses, performed in different spots on the surface of the materials (for further details refer to Figure S1 and Table S1 in the Supplementary Materials).

**Figure 4:**
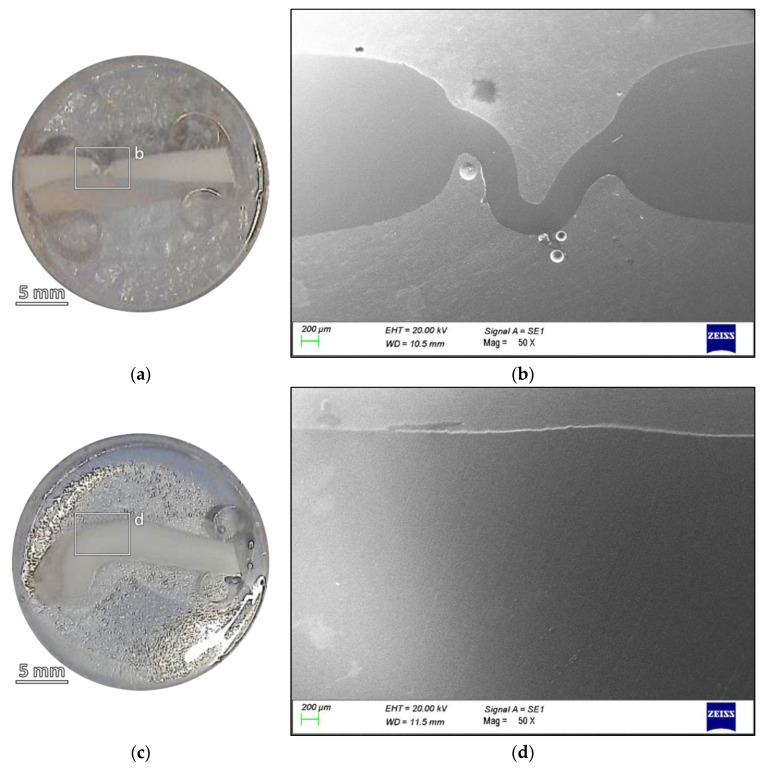
(**a**) Photograph and (**b**) SEM micrograph of the section of a Failed sample compared with (**c**) the photograph and (**d**) the SEM micrograph of the section of a Cured specimen.

In Figure 4a,c, cross-sectional photographs of liners are shown, from the Failed and the Cured sample, respectively. The portion of the liners are those close to the pinch-off line, where the decreased thickness and the cracks are observed; the same piece is cut from the Cured sample. The SEM micrographs, corresponding to the areas highlighted in the photographs, can be seen in Figure 4b,d.

By comparing the two samples, the plastic deformation that occurred in the Failed sample is again confirmed, and a strong reduction in the cross section is observable. Moreover, it is possible to see how the thickness reduction was followed by a bending deformation that very plausibly led to the fracture initiation.

**Figure 5:**
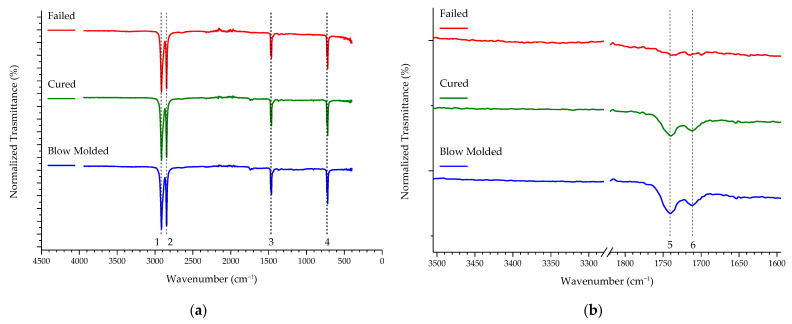
ATR-FTIR spectra of the analyzed samples: (**a**) full range of the spectra and (**b**) two specific ranges at ten-fold magnification, compared to the first panel.

The results of the FTIR analyses can be seen in Figure 5. The peaks recorded in the spectra, well identifiable in Figure 5a and listed below in Table 2, are those typical of PE [33,34] and display no significant variations between the different samples. The possible oxidation of the Failed sample, observed by EDXS, could not be undeniably confirmed from a first inspection of the spectra. Indeed, as can be seen in Figure 5b, which contains an extract of Figure 5a with a ten-fold magnification in the vertical scale, the peaks for the carbonyl group (C=O) in the carboxyl group (–C(=O)–OH), with a wave number of 1741 cm^−1^, are decreasing in intensity in the Failed sample. A weaker, but similar development is observed when focusing on the peak at 1711 cm^−1^, which is associated with C=O stretching vibration. In addition, in Figure 5b, a portion of the spectra centered on the wavenumber 3400 cm^−1^ is shown, as well, in a range usually associated with hydroperoxides, also involved in the oxidation of polyethylene. In this instance, no peak and no change in the background could be detected.

**Figure 6:**
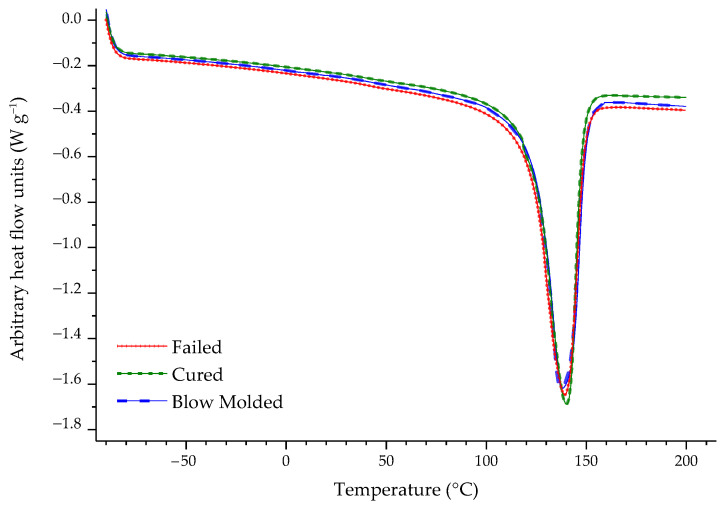
DSC profiles of all the analyzed samples.

The DSC profiles are reported in Figure 6 and their shape and aspect, with overlapping curves and extremely similar main features, suggests that almost negligible differences in the results derived from the thermograms are to be expected. These important parameters are reported in Table 3, which lists the data obtained through DSC analysis, specifically the values of melting enthalpy, crystallinity, onset temperature, and peak temperature. As can be read in Table 3 and also observed from the graphs in Figure S2, there are no significant differences among the calculated parameters.

**Figure 7:**
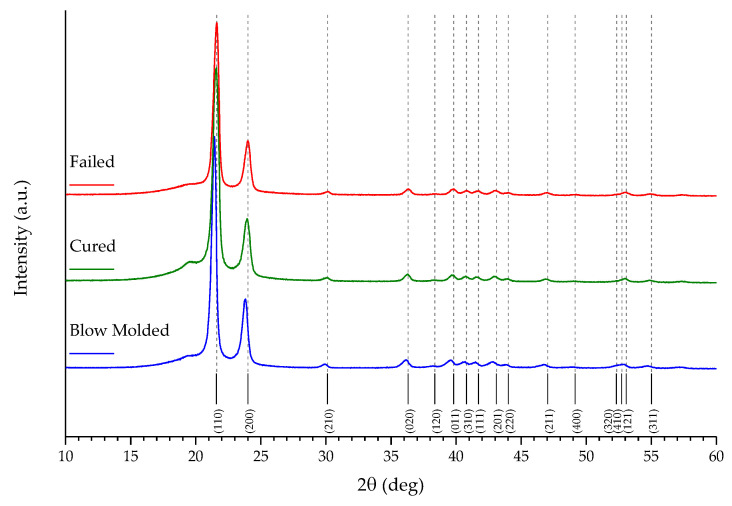
XRD patterns of the analyzed samples, with labels and notations for the Miller indices of the main lattice planes.

The XRD patterns of the three samples are reported in Figure 7. The three lines do not display remarkable differences and are in good agreement with each other and also with the reference [35].

**Figure 8:**
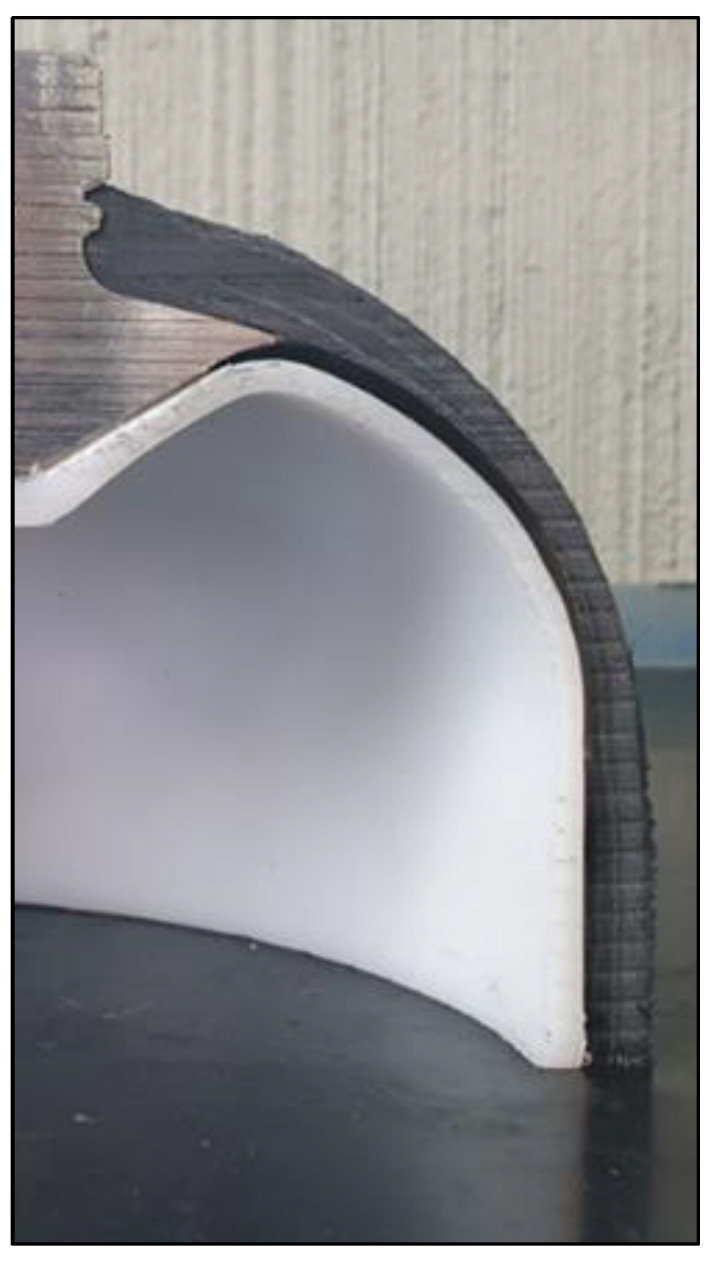
Section of the cured high-pressure storage tank.

A section of a Cured Type IV cylinder was cut for observation and the result can be seen in Figure 8. The picture shows that the metal boss is glued to the HDPE liner. At the end of the boss, the composite material shell should be well adhered to the liner, but a gap of a few millimeters can be seen between the composite itself and the polymer.

## 4. Discussion

As shown in Figure 1, the liner failed in the hemispherical head area, causing cracks to form perpendicularly to the mold parting line, on both the sides, with similar sizes, morphological characteristics, and features. The appearance of the area surrounding the cracks clearly suggests plastic deformation, a reduction of the thickness of the liner, the formation of oriented variation of opacity in the thinner areas, and a flow of deformed material pushing outward, presumably a few moments before the crack initiated. A closer look at the area where the failure occurred (*cf.* Figure 2) confirmed a severe plastic deformation, probably due to the prescribed standard test loading and unloading cycles. The characterization technique and the magnification used for the micrographs do not allow the discernment of macromolecular orientation, but the fiber fraying visible in Figure 2c and the changes to the surface morphology with the characteristic texture and rugosity observable in Figure 2d, suggest the formation of craze-like features in the material.

A comparison of the failure morphology with the surfaces of samples from different stages of the production process (*cf.* Figure 3) showed that there were no major changes in the surface morphology and its texture before the test. Figure 4, on the other hand, showed that one of the most plausible causes of the failure was a significant reduction in cross-sectional area compared to the Cured sample. This thinner portion was obviously more prone to yielding and failure, due to the combined effect of the high pressure reached during the loading phase of the cyclic test with mechanical fatigue and the concentration of the stress in the already deformed area, which is clearly visible in Figure 4b.

Therefore, an attempt was made to understand what the cause of this phenomenon was, from a material perspective, considering that the failure did not occur in similar specimens. However, it should be kept in mind that due to the industrial nature of the investigation, some information is confidential and could not be disclosed or even simply retrieved, to help differentiate different polymer batches and identify those prone to failure. A chemical analysis of the samples by EDXS shown in Table 1 showed the slight oxidation of the Failed sample, although this could not be detected by FTIR, as well. The values on the surface, near to the damaged area (*cf.* Figure S1a and Table S1, in the Supplementary Materials) are higher than those of the other samples, but those on the section, in the deformed material (*cf.* Figure S1d and Table S1), are more aligned with the Blow Molded and Cured sample values. Since the surface was conserved and exposed to air for much longer than the section, which was observed shortly after the cut and the sample preparation, the effect of oxygen from the atmosphere and from the high-pressure fluid during the cyclic test would have been stronger in this case.

In fact, considering spectrometric analyses, no noticeable oxidation or other difference among the different samples used was detected. In particular, the peaks for the carbonyl group (1741 cm^−1^ and 1711 cm^−1^) barely show in the spectra and went unnoticed in the noise (*cf.* Figure 5a). Nonetheless, the decreased intensity in the Failed sample (Figure 5b) would suggest the opposite of an increase in oxidation in the polymer. While the extent of oxidation measured with EDXS was not confirmed by FTIR analysis (*cf.* Figure 5), a minor oxidation phenomenon is entirely possible. Fatigue, failure, and subsequent bond breaking may have in fact caused the reaction with oxygen. In fact, while the phenomenon of oxidative degradation of PE usually occurs after heating in the molten polymer, it is not possible to exclude the circumstance of the auto-oxidation of the solid PE, happening at relatively low temperatures [36]. For instance, it could be caused by factors such as the presence of structural defects, or rapid heating. The exposure of a surface to oxygen can also facilitate the phenomenon, obviously. Even the interaction with high pressure hydrogen is among the causes for the formation of oxidized groups, including carbonyl groups in ester compounds [29].

The results of the DSC analysis (*cf.* Table 3 and Figure S2) confirmed that there are no major differences among the Blow Molded, Cured, and Failed samples. It has already been pointed out that the DSC profiles themselves (displayed in Figure 6) are practically superimposable and they do not differ in any peculiar aspect. Since there were no changes in the calculated parameters, *i.e.*, melting enthalpy, crystallinity, onset temperature, and peak temperature, the possibility of the thermal processes implemented on the HDPE liner, including the autoclave transition (needed for the composite shell material resin curing), affecting the polymer properties in any way could be ruled out.

The XRD patterns (*cf.* Figure 7) reinforce, with the presence of several sharp peaks, the data for the percentage of crystalline phase in the sample. After noticing the good alignment of all the peaks with the reference (for HDPE, an orthorhombic crystal structure with a *Pnam* space group [35]), it can be assessed that the peak width is similar in all three samples (*cf.* Table S3), meaning that the cell parameters are not distorted by any of the steps of the manufacturing or the subsequent testing. The amorphous phase is represented by the bump at around 20° and it can be better noticed in the expanded graphs in Figure S3, focusing on the range 10° < 2θ < 40°. In this range, by resolving the X-ray diffraction patterns, an estimation of the crystallinity index has been evaluated from the diffraction area relative to the crystalline peaks compared to the one relative to the amorphous halo [37,38]. The percentages of crystalline phase are reported in Table S2 and are in agreement with the results calculated from the DSC profiles and displayed in Table 3; estimations from the XRD patterns, however, may be slightly distorted from the cutting of the specimen, which could deform and overheat some layers of the analyzed samples. The detailed graphs in Figure S3, together with the data reported in Table S3, allow, in addition, a better observation of the position of the peaks, which in the case of the Blow molded sample, are to some extent shifted left, suggesting a minuscule deformation/expansion of the cells. One hypothesis to explain this evidence is the minor effect of the manufacturing process that then disappears with the curing procedure and is, therefore, absent in the other two samples.

Since the collected results and data allow us to exclude the possibility that the thinning of the liner section is due to chemical/physical phenomena occurring during the manufacturing process, it can be assumed that the cause is ascribable to design reasons. As shown in Figure 8, the adhesion between the liner and the metal boss is provided by an adhesive, sealing these two parts without leaks or gaps [39]. On the other hand, the adhesion between the composite shell and the liner is not perfect. Therefore, it can be hypothesized that loading and unloading cycles, combined with a heterogeneous stress distribution in the transition area between the boss contact and the composite material, cause a combination of stress phenomena such as plastic deformation, local heating [40], and void formation [41] in the polymer, resulting in section thinning.

## 5. Conclusions

In this paper, a comprehensive failure analysis was conducted on a liner from a Type IV high-pressure gas storage cylinder. The failure had occurred in the hemispherical head area during a cyclic pressure test designed to check the tightness of the cylinder. The results of the analysis showed that:

- The failure was caused by strong plastic deformation resulting in a decrease in cross-section thickness.
- Morphological and chemical analyses performed by means of SEM/EDXS, FTIR, XRD, and DSC did not show any major changes occurring during the production cycle of the liner, which could justify the creation of a trigger point for failure.
- The reason for the failure was plausibly due to a geometric issue. In a fatigue test with pressure cycles, the physical gap between liner and shell causes small deformations that can lead to the observed thickness reduction. The improvement of the interface between liner and shell, for example with the use of improved adhesives, could solve or mitigate the issue.

## Figures and Tables

**Table 1:** Surface chemical composition of the analyzed samples.

Sample	% C	% O
Blow Molded	96.64 ± 0.47	3.36 ± 0.47
Cured	97.58 ± 1.20	2.42 ± 1.20
Failed	93.89 ± 1.06	6.11 ± 1.06

**Table 2:** Assignment of the FTIR bands visible in Figure 5.

Bands	Wavenumber (cm^−1^)	Assignment
1	2919	CH_2_ asymmetric stretching
2	2851	CH_2_ asymmetric stretching
3	1473 and 1463	Bending deformation
4	731 and 720	Rocking deformation
5	1741	C=O
6	1711	C=O

**Table 3:** Data calculated from the analysis of the DSC profile visible in Figure 6.

Sample	Melting Enthalpy (J g^−1^)	Crystallinity (%)	T _onset_ (°C)	T _peak_ (°C)
Blow Molded	166.417 ± 4.853	56.798 ± 1.656	123.653 ± 0.294	136.500 ± 2.548
Cured	160.317 ± 3.211	54.716 ± 1.096	123.550 ± 0.918	134.340 ± 1.011
Failed	169.600 ± 0.269	57.884 ± 0.092	123.270 ± 0.679	132.840 ± 0.552

## Data Availability

The data presented in this study are openly available in Zenodo at https://doi.org/10.5281/zenodo.10624669.

## Supplementary Materials

The following supporting information can be downloaded at: https://www.mdpi.com/article/10.3390/polym16060779/s1; Figure S1: SEM micrographs with details of the areas where the EDXS analyses were performed; Table S1: surface chemical composition of the portion of samples analyzed; Figure S2: data calculated from DCS analysis reported in graphical form; Figure S3: XRD patterns in the 10–40° range, with the integrated area shown, of all the samples; Table S2: estimated percentage of crystalline phase from the integrated area in Figure S3; Table S3: detailed data retrieved from diffraction peaks of the most significant reflections reported in Figure S3.
