# Supplementary material for: Study of the Failure Mechanism of a High-Density Polyethylene Liner in a Type IV High-Pressure Storage Tank
Source: Polymers (Basel). 2024 Mar 12;16(6):779. doi: 10.3390/polym16060779 (PMC10975935; doi:10.3390/polym16060779)
Supplement: Supplementary file 1 [file polymers-16-00779-s001.zip › polymers-2889073-supplementary.pdf]

Supplementary Materials

Study of the Failure Mechanism of a High-Density Polyethylene Liner in a Type IV High-Pressure Storage Tank

Alfredo Rondinella [1,*], Giovanni Capurso [1,*], Matteo Zanocco [1], Federico Basso [2], Chiara Calligaro [3], Davide Me-notti [3], Alberto Agnoletti [3], Lorenzo Fedrizzi [1]

[1] Polytechnic Department of Engineering and Architecture, University of Udine, via del Cotonificio 108, 33100 Udine, Italy; matteo.zanocco@uniud.it (M.Z.); lorenzo.fedrizzi@uniud.it (L.F.)

[2] Department of Agricultural, Food, Environmental, and Animal Sciences, University of Udine, via Son-drio 2/A, 33100 Udine, Italy; federico.basso@uniud.it (F.B.)

[3] Faber Industrie SpA, Via dell'Industria 64, 33043 Cividale del Friuli, Italy

* Correspondence: alfredo.rondinella@uniud.it (A.R.); giovanni.capurso@uniud.it (G.C.)

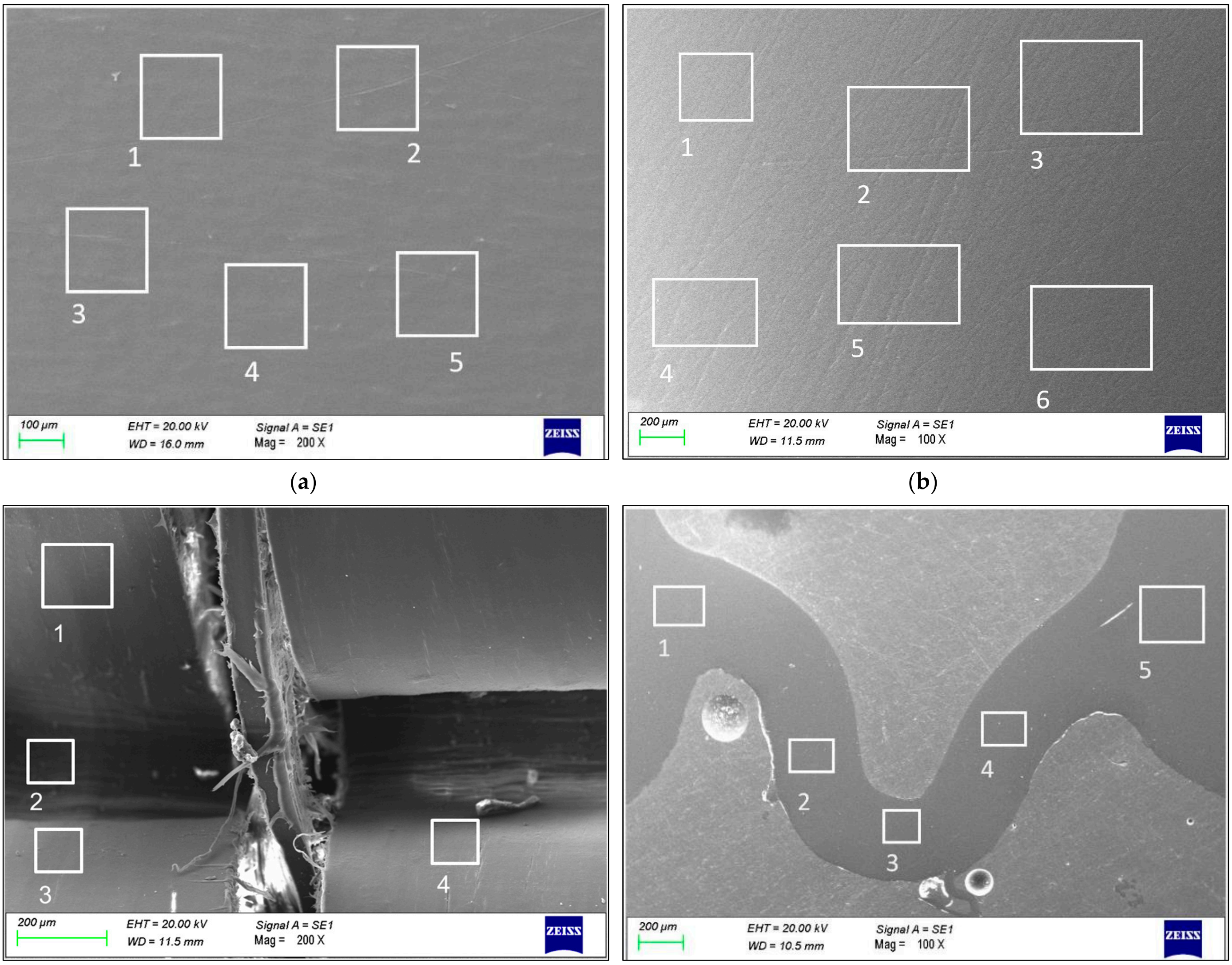

(**a**) (**b**) (**c**) (**d**)

Figure S1. SEM micrographs with details of the areas where the EDXS analyses were performed (with labeling reported in Table S2) of: (**a**) the Blow Molded sample, (**b**) the Cured sample, (**c**) the

Failed sample, in the area close to one of the cracks highlighted in Figure 2, and (**d**) the Failed sample cross section, as shown in Figure 3.

Table S1. Surface chemical composition of the portion of samples analyzed, as indicated and labeled in Figure S2.

Sample	Label	% C	% O
Blow Molded	(**a**) 1	96.08	3.92
	(**a**) 2	96.49	3.51
	(**a**) 3	97.25	2.75
	(**a**) 4	96.25	3.75
	(**a**) 5	97.13	2.87
Cured	(**b**) 1	96.25	3.75
	(**b**) 2	97.00	3.00
	(**b**) 3	97.90	2.10
	(**b**) 4	100.00	0.00
	(**b**) 5	97.50	2.50
	(**b**) 6	96.84	3.16
Failed (surface)	(**c**) 1	92.14	7.86
	(**c**) 2	94.88	5.12
	(**c**) 3	94.00	6.00
	(**c**) 4	94.54	5.46
Failed (section)	(**d**) 1	95.22	4.78
	(**d**) 2	96.73	3.27
	(**d**) 3	97.08	2.92
	(**d**) 4	98.07	1.93
	(**d**) 5	96.43	3.57

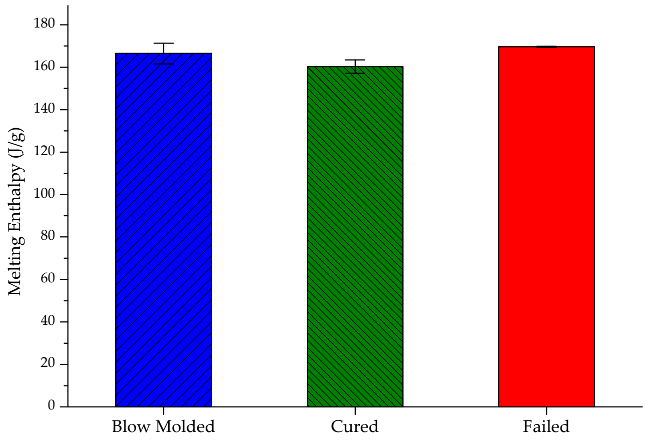

(**a**)

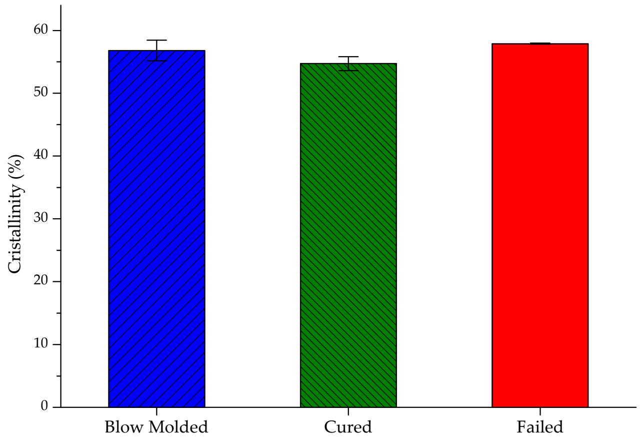

(**b**)

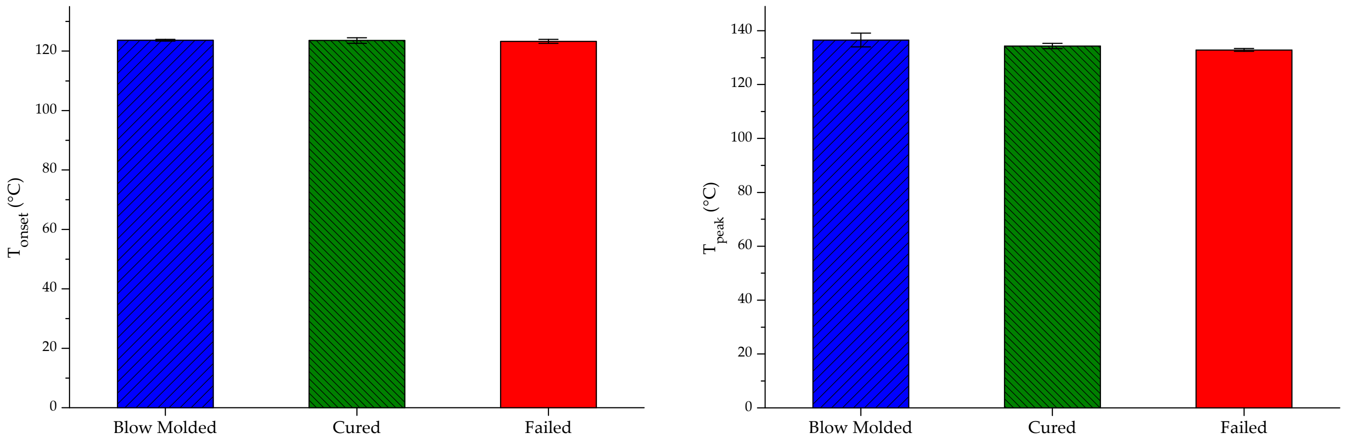

(**c**) (**d**)

Figure S2. Data calculated from DCS analysis reported in graphical form: (**a**) Melting Enthalpy, (**b**) Crystallinity, (**c**) Onset Temperature, and (**d**) Peak Temperature.

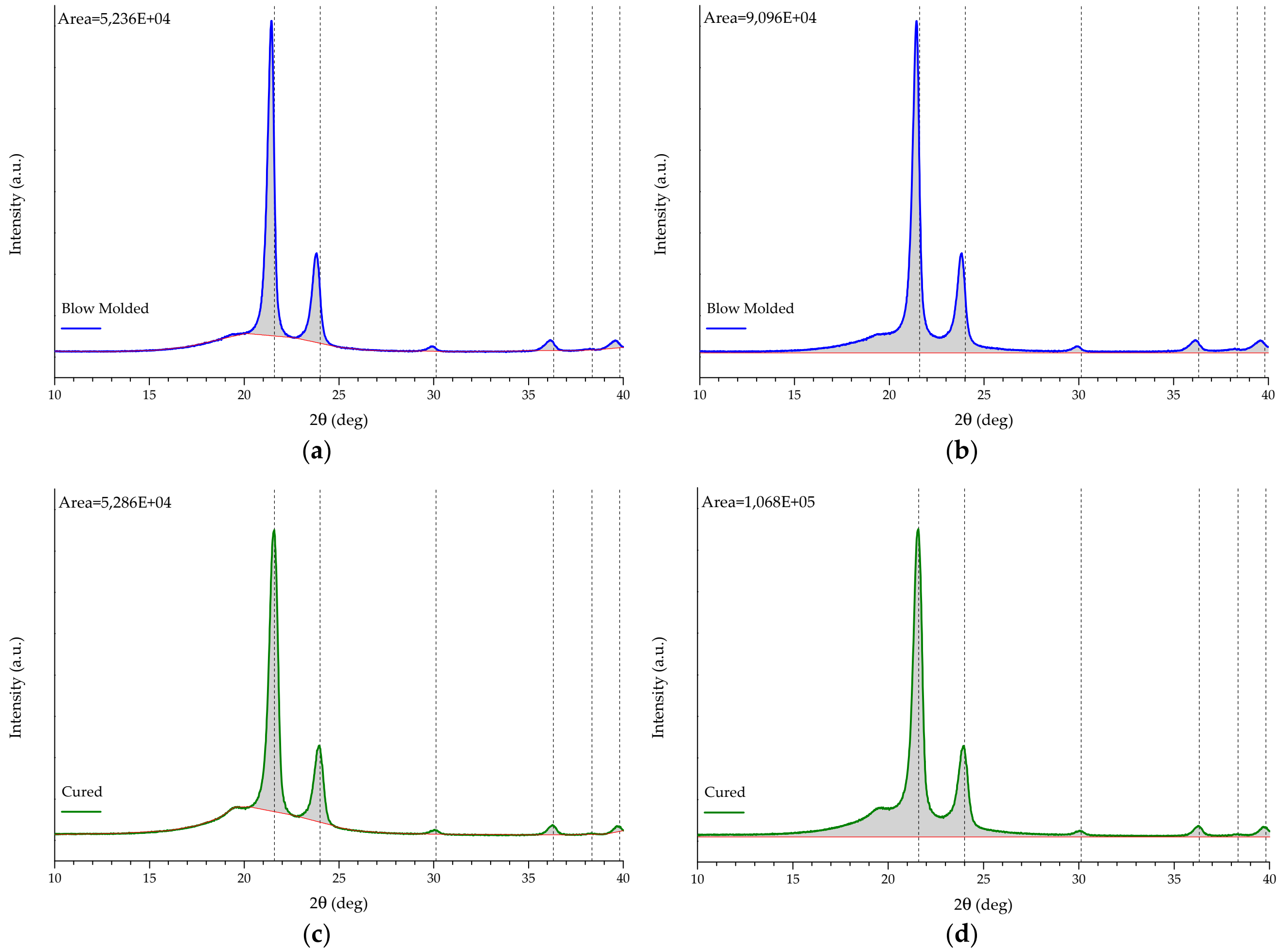

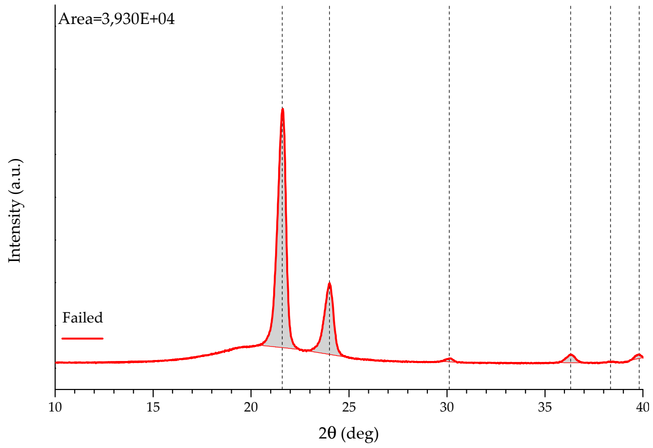

(e)

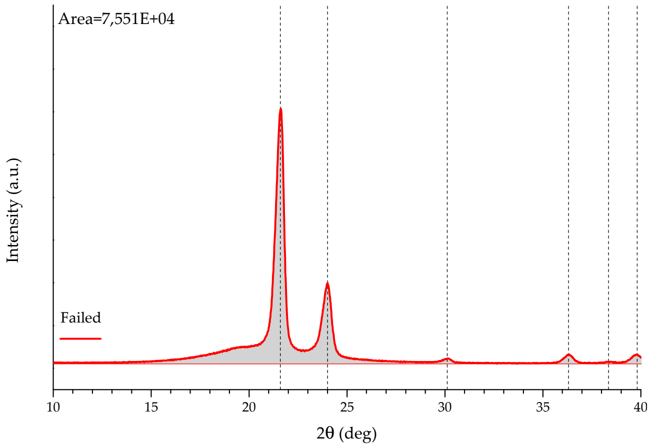

(f)

Figure S3. XRD patterns in the 10–40° range, with the integrated area shown: (**a**) peaks area and (**b**) total area for the Blow Molded sample; (**c**) peaks area and (**d**) total area for the Cured sample; (**e**) peaks area and (**f**) total area for the Failed sample.

Table S2. Estimated percentage of crystalline phase from the integrated area in Figure S3.

Sample	Peaks area	Total area	Est. crystalline phase
Blow Molded	52356.076	90963.366	57.55 %
Cured	52861.129	106766.476	49.51 %
Failed	39299.435	75512.561	52.04 %

Table S3. Detailed data retrieved from diffraction peaks of the most significant reflections reported in Figure S3.

Reflection	Sample	Diffraction angle (deg)	FWHM (deg)	Intensity (a.u.)
(110)	Blow Molded	21.445	0.40341	81280
	Cured	21.581	0.52294	75070
	Failed	21.632	0.47418	60695
(200)	Blow Molded	23.825	0.47729	25079
	Cured	23.944	0.55787	22919
	Failed	24.012	0.51076	19888
